# Genome Sequence of the Bile Salt-Degrading Bacterium *Novosphingobium* sp. Strain Chol11, a Model Organism for Bacterial Steroid Catabolism

**DOI:** 10.1128/genomeA.01372-17

**Published:** 2018-01-04

**Authors:** Onur Yücel, Daniel Wibberg, Bodo Philipp, Jörn Kalinowski

**Affiliations:** a University of Münster, Institute of Molecular Microbiology and Biotechnology, Münster, Germany; b Center for Biotechnology (CeBiTec), Bielefeld University, Bielefeld, Germany

## Abstract

Many bacteria from different phylogenetic groups are able to degrade eukaryotic steroid compounds, but the underlying metabolic pathways are still not well understood. *Novosphingobium* sp. strain Chol11 is a steroid-degrading alphaproteobacterium. Its genome sequence reveals that it lacks several genes for steroid degradation known to exist in other model organisms.

## GENOME ANNOUNCEMENT

Bile salts are surface-active steroid compounds with diverse functions in vertebrates, e.g., in digestion of lipophilic nutrients in the intestinal tract (1, 2). These steroids are released by fecal and urinal excretion into the environment, where they can serve as a source of carbon and energy for many soil and aquatic bacteria (3, 4). Bile salt degradation has been studied mainly with *Pseudomonas stutzeri* strain Chol1, *Comamonas testosteroni*, *Rhodococcus jostii*, and *Pseudomonas putida* (5–8) and involves very similar metabolic pathways in all of these model organisms. However, some bacteria were shown to initiate the degradation of 7α-hydroxy-bile salts by an alternative pathway (3). Among these is the alphaproteobacterium *Novosphingobium* sp. strain Chol11, which has been isolated from freshwater. Degradation of the bile salt cholate is initiated by the 7α-dehydratase Hsh2 leading to metabolites with a 3-keto-∆^4,6^-diene structure of the steroid skeleton (9). The steroid skeleton of these metabolites cannot be degraded by, e.g., *P. stutzeri* strain Chol1 (4, 9). For further investigating this potential novel pathway for bile salt degradation, the genome of *Novosphingobium* sp. strain Chol11 was sequenced.

Genomic DNA was purified with the Puregene tissue core kit B (Qiagen). Purified DNA was used to construct an 8-kbp mate pair sequencing library (Illumina, USA). Assembly of the obtained and processed reads was performed by applying GS *de novo* Assembler software version 2.8 (Roche, Mannheim, Germany). Assembly resulted in 52 contigs and 10 scaffolds. *Novosphingobium* sp. strain Chol11 scaffolds were mapped onto the *Sphingobium chloroplenolium* L-1 (GenBank accession no. CP002798 to CP002800, BioProject no. PRJNA224116) reference replicons by means of r2cat (10), followed by an *in silico* gap closure approach (11). Annotation of the draft replicons was performed within the GenDB2.0 system (12) and Prokka version 1.11 (13).

The Chol11 draft genome comprises two chromosomes and two plasmids. Chromosome 1 (2.54 Mb) and chromosome 2 (0.86 Mb) encode 2,494 and 776 open reading frames (ORFs), respectively. Together with the plasmids pSa (circular, ca. 0.13 Mb) and pSb (linear, 0.13 Mb), the total genome size is about 3.66 Mb and encodes 3,532 putative proteins. The mean GC content is 62.44%.

Most of the genes with a potential role in steroid degradation are located on chromosome 2, including *hsh2*. Compared to the genome of, e.g., *Pseudomonas stutzeri* Chol1 DSM 103613 (14), several genes involved in the degradation of the steroid side chain do not have orthologues in strain Chol11. Thus, the genomic content supports the presence of an unexplored pathway for bile salt degradation in *Novosphingobium* sp. strain Chol11.

### Accession number(s).

The scaffolds of the whole-genome shotgun project have been deposited in the EMBL database (EBI) under the accession no. OBMU01000001 to OBMU01000010.
